# The High Obesity Program: A Collaboration Between Public Health and Cooperative Extension Services to Address Obesity

**DOI:** 10.5888/pcd17.190283

**Published:** 2020-03-19

**Authors:** Sahra A. Kahin, Ashleigh L. Murriel, Anu Pejavara, Terrence O’Toole, Ruth Petersen

**Affiliations:** 1 Centers for Disease Control and Prevention, Atlanta, Georgia

In the United States, obesity is a major risk factor for chronic disease, and related medical costs are estimated to increase by at least $48 billion annually through 2030 (1). Interventions that use policy, systems, and environmental (PSE) approaches at the population level, such as increasing the availability of healthy foods in local corner stores or incorporating activity-friendly routes into community planning and design, can expand the reach of public health efforts by establishing frameworks in which the simple, default choices are the healthier choices in the places Americans work, live, and play (2).

The Centers for Disease Control and Prevention (CDC) is committed to improving the health of Americans through evidence-based public health programs; the agency supports these programs through funding mechanisms called cooperative agreements that are awarded to state and local public health entities. A cooperative agreement provides for substantial involvement between a federal awarding agency and a nonfederal entity in carrying out defined activities. This editorial describes activities designed to strengthen partnerships to improve health through PSE approaches.

In 2014, CDC’s Division of Nutrition, Physical Activity, and Obesity launched a program called Programs to Reduce Obesity in High Obesity Areas, also referred to as HOP. The program was a result of congressional funding authorization for land-grant universities (LGUs) to work with the US Department of Agriculture’s Cooperative Extension Services (CES) to launch an outreach program to combat obesity where obesity rates are the highest.

From 2014 through 2018, CDC’s HOP provided funding to 11 LGUs in states with counties in which the prevalence of adult obesity was greater than 40% according to data from the 2013 Behavioral Risk Factor Surveillance System. CDC staff members provided substantive guidance to the LGUs through program support from CDC project officers and evaluators. These CDC staff members have expertise in HOP areas and provided technical assistance and guidance to LGUs on evidence-based nutrition and physical activity interventions, community-based participatory approaches, community needs assessments, coalition development, performance measures, and leveraged resources (eg, financial, in-kind donations, volunteer hours, additional grant funding). CDC provided this expertise and technical assistance through monthly calls, work plan reviews, and community site visits. LGUs provided direct support and guidance to their respective CES to conduct evidence-based nutrition and physical activity interventions in eligible counties.

HOP uses the knowledge and relationships of CES and communities to improve the nutrition and physical activity environments in primarily rural counties. CES aims to “advance agriculture, the environment, human health and well-being, and communities” (3) by supporting research, education, and extension programs in the LGU system and other organizations. HOP funding supported and facilitated LGUs’ and CES’ expansion of focus to also include PSE as an approach to obesity interventions and strategies. Working with CES is a benefit for CDC because CES agents have established relationships with partners in the communities in which they work and an intimate knowledge of assets and needs in those communities.

HOP recipients used a community-based participatory approach during the first 6 months to 1 year of the cooperative agreement to engage community coalitions and conduct community needs assessments. HOP recipients worked on the following 3 strategy approaches:

1. Providing education and promotional support for environmental approaches.
2. Implementing evidence-based practices to increase consumption of healthy foods and beverages.
3. Implementing evidence-based strategies to increase opportunities for physical activity.

On the basis of CDC guidance, HOP recipients elected to work in either the community or the early care and education setting.

The purpose of this collection of articles related to HOP in *Preventing Chronic Disease* is to highlight the program’s approach and describe both overarching and program-specific evaluation findings. The collection comprises 8 articles, 7 that highlight the work of LGUs (4–10), and one that describes HOP’s implementation approach, evaluation framework, and key findings (11). 

Powers and colleagues described a 9-week, multilevel, faith-based health promotion initiative that used PSE approaches in 14 Alabama faith communities (4). A one-group pretest–posttest study evaluated faith community policies and environments, interpersonal support, and individual behaviors. Seventy-two sessions with 737 adults were implemented in 14 faith communities. Participants in the small group sessions reported feeling more supported to engage in healthy eating behaviors. The authors outlined an approach that faith communities can use to support and evaluate healthy lifestyles.

Carter and colleagues summarized findings from a community-based obesity reduction and prevention initiative implemented to increase opportunities for physical activity among residents in rural Alabama (5). This initiative worked with 14 community coalitions to implement 101 interventions related to physical activity throughout 16 communities. To better assess community needs and areas to implement a community-based obesity intervention, the authors conducted focus groups with each of the coalitions. They explained how the use of a community-based participatory research approach may be an effective way to identify and address health concerns at the local level.

Gustafson and colleagues examined the effectiveness of community-based interventions implemented in rural Kentucky (6). They reported findings from a random-digit–dialing cross-sectional survey from 2 random samples of adult residents in 6 participating rural counties before and after community-based interventions were implemented. From year 1 to year 2 of the intervention, fruit and vegetable intake significantly increased; moderate physical activity, as measured in days per week, did not significantly change; and attitudes among residents about places to be physically active improved. The findings illustrate how community involvement in promoting obesity prevention initiatives may have a significant effect on dietary intake and community perception about places to be physical active.

Kendall and colleagues described findings from a community-based project implemented in 3 rural Louisiana parishes that focused on promoting healthy eating and physical activity through PSE approaches (7). After conducting coalition assessments, the initiative implemented multipronged interventions in 5 food stores across the participating parishes. This community-based project identified several important factors to consider when implementing environmental rural food interventions: store size, owner preferences, distributor contracts, in-store marketing, and intervention strength.

Stluka and colleagues examined collaborations with residents of rural communities in South Dakota to implement and evaluate garden-based interventions (8). The authors reported that 13 gardens were established through 18,136 hours of volunteer work. Evaluation findings showed that an average of 138 pounds of food were harvested per garden site. The authors indicated that the implementation of community gardens could generate substantial amounts of produce and provide opportunities for collaboration among local community members and organizations.

Wallace and colleagues described an initiative implemented in 4 rural western counties in Tennessee that engaged community residents in activities to reduce obesity and used a PSE framework and a community-based participatory approach (9). Evaluators conducted various assessments (focus groups, audits, pedometer monitoring, and mapping) to determine the number of community members potentially served as a result of the initiative and how the initiative affected attitudes and behaviors. The authors reported improvements in physical activity and healthy eating among participating community members.

Castillo and colleagues described how needs assessments were used to identify components of a PSE-centered initiative implemented in 4 communities in Hidalgo County, Texas, to increase access to physical activity and healthy foods (10). The needs assessments identified gaps in active living infrastructure for physical activity and recommended individuals to help establish local community coalitions. The program successes demonstrated that community-driven PSE interventions can be a strategy in establishing long-term solutions for obesity prevention.

This special collection in *Preventing Chronic Disease* describes approaches to improve the nutrition and physical activity environments in rural areas that have a high prevalence of adult obesity. Articles in this collection support the approaches of previous studies on interventions to improve health outcomes, such as the use of tailored community-based participatory approaches and a focus on using PSE when improving the nutrition environment and opportunities for physical activity in communities. The collection provides examples of community interventions that aim to increase the healthfulness of food and access to physical activity, such as improving healthy food options in retail outlets (7), creating opportunities for physical activity through local organizations (5), and collaborating with nontraditional public health partners, such as CES (4–10). The approaches described in this collection may provide organizations and community-based programs ideas for implementation of future work to improve the nutrition and physical activity environments in rural areas with a high prevalence of obesity.

The findings from HOP influenced the approach and expectations of the subsequent HOP funding period, which began in 2018, and other cooperative agreements funded by CDC. CDC used the emerging approach of collaboration with CES in its current cooperative agreements. CDC continues in its expectation that state and local recipients engage coalitions through community-based participatory approaches, use the results of community needs assessments to drive the selection of interventions, and tailor approaches to meet the unique needs of priority populations and communities. Other funding organizations addressing obesity may consider these approaches for implementation of future community-level work.
